# Maximising camera trap data: Using attractants to improve detection of elusive species in multi-species surveys

**DOI:** 10.1371/journal.pone.0216447

**Published:** 2019-05-29

**Authors:** David Mills, Julien Fattebert, Luke Hunter, Rob Slotow

**Affiliations:** 1 School of Life Sciences, University of KwaZulu-Natal, Durban, South Africa; 2 Panthera, New York, NY, United States of America; 3 Department of Genetics, Evolution and Environment, University College, London, United Kingdom; University of Sydney, AUSTRALIA

## Abstract

Camera traps are a key tool in ecological studies, and are increasingly being used to understand entire communities. However, robust inferences continue to be hampered by low detection of rare and elusive species. Attractants can be used to increase detection rates, but may also alter behaviour, and little research has evaluated short-term, localized response to the presence of attractants. We conducted three camera trap surveys in Kibale National Park, Uganda, using food baits and scent lures (“attractants”) at each camera station to entice small carnivores to pass in front of camera stations. To examine the interrelationship between scavenging and response to attractants, we also placed camera traps at five food refuse pits. We modelled the effect of attractant and duration of trap placement on the detection probability of small carnivores and selected African golden cat *Caracal aurata* prey items. We examine transient site response of each species, by comparing our observed likelihood of detection in each 24 h period from 1–7 d following refreshing of attractants to randomly generated capture histories. African civet *Civettictis civetta*, rusty-spotted genet *Genetta maculata*, African palm civet *Nandinia binotata*, and marsh mongoose *Atilax paludinosus* detection probabilities were highest and Weyns’s red duiker *Cephalophus wenysi* detection probability was lowest immediately after attractants were placed. Within 24 h after attractant was placed, rusty-spotted genet and African palm civet were more likely to be detected and African golden cat, red duiker, and blue duiker *Philantomba monticola* were less likely to be detected. Our results suggest that attractants can increase detection of small-bodied species and include some arboreal species in terrestrial camera trap sampling. However, attractants may also alter short-term visitation rates of some species, with potentially cascading effects on others. Community level and intraguild interaction studies should control for the potentially confounding effects of attractants on spatial activity patterns.

## Introduction

Remotely triggered camera traps are essential tools in the study of rare and elusive species [1–3]. Technology and analysis techniques for camera-trap data [4–6] in biodiversity monitoring [7, 8], regional occupancy and species richness surveys [9, 10], and single species and community ecology studies [11–14] have evolved rapidly. Because many threatened species have extremely low detection probabilities [3, 15, 16], it is common practice to increase sample sizes and ensure statistically robust results by placing cameras at species-preferred sites [16–19] or extending survey duration [20, 21]. As current conservation efforts shift away from focusing on large, charismatic species to include smaller species that have not traditionally been the primary focus of research [1, 14], the problem of limited sample size is further compounded by naturally lower detection probabilities of small bodied species [22], and the influence of interference competition from larger predators [23]. Increased sample sizes of species with low detection probabilities can be achieved by expanding survey area or extending survey length, but these can come with substantially increased investments of time and money [24, 25].

Placing attractants (edible baits or inedible lures) at camera stations increases capture rates for some species [26–28]. However, using attractants remains controversial because it may also introduce behavioural and statistical biases resulting from a violation of geographic closure assumptions, changes in activity patterns, and habituation to attractant stations [28–31].

Understanding species-specific and time-varying response to attractants would inform the design and analyses of camera trap surveys using attractants, and allow more robust interpretation of the resulting data with respect to inter-specific community interactions [14, 32]. An assessment of several attractant biases suggested that, at least for small carnivores, their effects may be minimal [28]. Several studies have studied the impacts of attractants using unbaited and baited stations in the same survey or stations that were unbaited for the first portion and baited for the second. These revealed the impacts of attractants on a broad scale spanning entire surveys [16, 33]. However, two aspects of attractant response remain poorly understood. First, there is limited knowledge of how attractants alter camera station visitation through time as the potency (freshness) of attractants wane, specifically with respect to short-term changes in activity levels at camera stations. Second, not all species in a community are equally influenced by baits and lures, and some may actually be deterred [33].

We modelled the effect of attractant age on the detection probability of forest carnivores in south-western Uganda. Our primary purpose for using attractants in these surveys was to expand the scope of our sampling regime to include smaller species that had very low detection probabilities in previously surveys that did not use attractants (Mills, unpublished data) [22]. Preliminary tests revealed that African palm civets *Nandinia binotata* often descended trees to take meat and fruit from camera stations, which corresponds to previous research indicating they occasionally descend to the ground to feed [34]. Therefore, we capitalised on this behaviour in order to include this primarily arboreal species in our carnivore community study. We expected detection probability to change over time, either decreasing in the case of scavengers, or increasing if the placement of attractant led to initially reduced activity the station. Of primary concern was whether or not the detection probability of some species, particularly prey species, was significantly reduced when attractant was fresh. We did not expect attractant to significantly increase the detection of larger carnivores in this community, such as African golden cats *Caracal aurata* (Temminck, 1827) and African civets *Civettictis civetta* (Schreber, 1776). We expected increased detection of smaller carnivores and decreased detection of prey immediately after attractant was placed and for this effect to subsequently decrease to background detection levels. We conclude by providing recommendations for the use of attractant in small carnivore studies.

## Methods

### Study area

**Fig 1:**
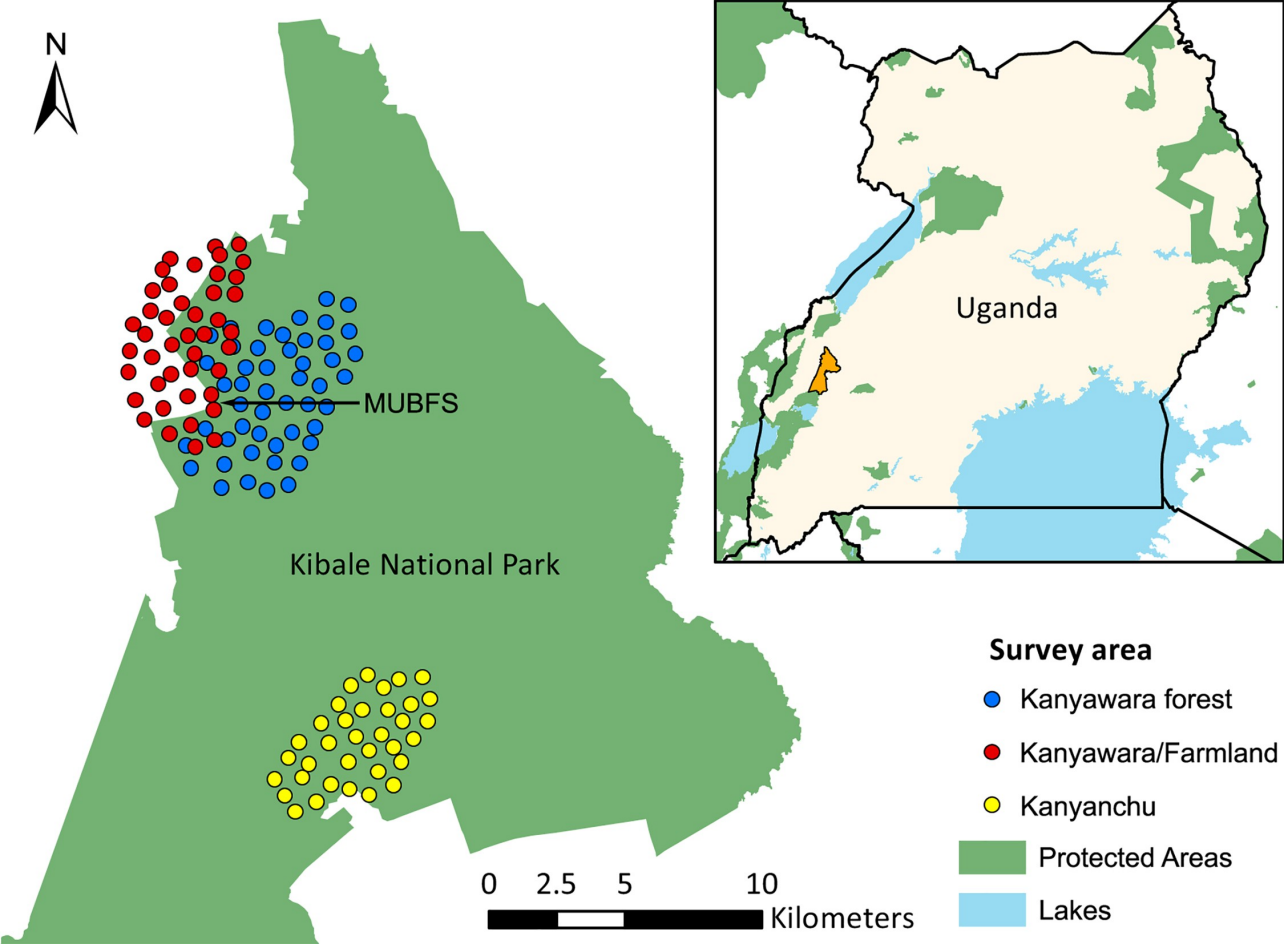
Camera locations from three camera trap surveys conducted in 2013–2014 Kibale National Park and adjacent farmland. Inset shows the location of the study area in western Uganda. The location of the Makerere University Biological Field Station (MUBFS) is indicated by the black arrow. (Protected Areas: UNEP-WCMC and IUCN (2018); Kibale boundary courtesy of Joel Hartter).

Kibale National Park is an isolated 795 km^2^ mid-altitude moist evergreen forest located in south-western Uganda. Kibale is a mosaic of tropical forest, regenerating forest, bush, grassland and papyrus swamp [35]. Two rainy seasons occur from March to May and September to November, with some annual variation (Chapman and Chapman, unpublished data). Mean annual rainfall is 1701 mm [36]. Average minimum and maximum temperatures are 15.5 C and 23.7 C [37]. Camera surveys were conducted in the Kanyawara research area in western Kibale, farmland to the west of Kanyawara, and in the Kanyanchu chimpanzee tourist area in the centre-east of the park (Fig 1).

### Study species

We tested the effect of attractants on carnivore species detected at least 30 times: African golden cat, serval *Leptailurus serval* (Schreber, 1776), African civet *Civettictis civetta*, African palm civet *Nandinia binotata* (Gray, 1830), servaline genet *Genetta servalina* (Pucheran, 1855), rusty-spotted genet *Genetta maculate* (Gray, 1830), and marsh mongoose *Atilax paludinosus* (G. Cuvier, 1829). We also tested the attractant response of three potential prey items of the African golden cat: blue duiker *Philantomba monticola* (Thunberg, 1789), Weyns’s red duiker *Cephalohpus weynsi* (Thomas, 1901), and giant forest rat *Cricetomys emini* (Wroughton, 1910) [38, 39].

### Camera placement

Attractants have been shown to impact species differently [33]. The presence of edible bait may increase detection probabilities of scavengers more than non-scavengers. We therefore investigated the interrelationship between attractants and scavenging behaviour. In order to determine which species in Kibale are regular scavengers, we deployed one camera at each of five organic rubbish pits at the Makerere University Biological Field Station. Kitchen food refuse discarded into these pits was entirely biodegradable and included meat, fruits, and vegetables. Cameras were placed so that animals would be photographed passing, entering, or exiting the pits.

To test the effects of attractants on the forest carnivore community, three systematic camera trap surveys were conducted in 2013 and early 2014 using 49, 43, and 35 camera stations respectively. We employed a grid-based layout with a spacing of 600–800 m (see SI 3). Since many carnivores preferentially use trails to navigate and patrol their territory [11, 17, 40], we searched within 200 m of designated camera positions to locate an animal or research trail that showed evidence of regular and recent use by small and medium sized mammals, as evidenced by a well-worn trail and fresh tracks. We installed two cameras at each station on opposite sides of the trail in order to capture both sides of each individual. To maintain continuity in detection probability, we deployed one Pantheracam v. 4 (Panthera, New York, NY) camera at each station. The second camera was another Pantheracam, Deercam DC300 (Non Typical Inc., Park Falls, WI), CamTrakker Ranger (CamTrak South Inc., Watkinsville, GA), Recon Digital Pro Scouting Camera (Recon Outdoors, Huntsville, AL), Stealth Cam HD (Stealth Cam, Grand Prairie, TX), or Cuddeback Capture IR (Non Typical Inc., Park Falls, WI). Cameras were installed 2 m from the trail centre and aimed 25 cm above the trail. Cameras were left in place for 60–90 days to ensure population closure [41].

### Attractant protocol

We used attractants to increase detection probability of small carnivores that were already active in the vicinity of the traps and that might otherwise walk around or run past the cameras. Our intent was to avoid altering habitat use of individuals, thereby biasing future analyses [21]. In order to minimise scent transmission and increase the likelihood of capture only for animals that were already active near camera stations, we placed all bait and scent lure on the ground [28]. An important goal of these surveys was to identify individuals of uniquely marked species. While fixed, inaccessible bait has been used in the past [28], we chose to use loose bait rather than fixing it in place. We did this to increase the likelihood that, within the same station visit (independent capture), individuals would present themselves at a variety of angles to the camera, and thus provide us with multiple options for confirmation of individual identification (D. Mills, unpublished data from pilot survey). In order to attract all species from the forest carnivore community, we created an attractant complex consisting of meat, fruit, and scent. We placed three pieces of beef to attract obligate carnivores and scavengers. We used two small sweet bananas cut into thirds to attract the primarily frugivorous African palm civet [42]. To reduce the chance of habituation to the station, and to allow time for the attractant effect at the station to wear off, the amount of edible bait placed was small enough to be fully consumed by a single individual in one encounter. We used two sprays of Calvin Klein Obsession for Men (CKO), which has been shown to elicit a response in felids [43], on a termite-eaten (porous) piece of wood, to attract African golden cats and non-scavengers. We refreshed attractant and serviced cameras once each week (mean: 8.15 ± 0.12 d; range: 3–27 d). This meant that the potency or freshness of the attractants decreased by desiccation or removal of meat or fruit, and dissipation of scent. Therefore, attractant potential at the end of the week was much lower than it was immediately after baiting. By comparing trends in detection probability and activity levels throughout the week, we were able to test for short-term biases caused by the use of attractants.

### Statistical analysis

We used an occasion length of 1 d for detection probability analyses. We investigated the transient effect of attractant freshness on detection using single-season occupancy models [41]. To test for the influence of attractant freshness on detection probability, we created an observation level covariate matrix indicating how many days had elapsed since attractant was refreshed. In order to compare presence of attractant with increasing effort, we tested two measures of survey effort: the total days each station was deployed (one value per station) and an observation level covariate matrix where the value for a given station at a given occasion was the number of days the camera station had been effectively active from the date the station was installed up to that occasion. This latter value ranged from one (day station was installed) to the total trap days accrued for that station. We kept occupancy constant (null model) and tested the influence of attractant and effort on detection probability both univariately and additively. Models were evaluated using the Akaike’s Information Criterion corrected for small sample sizes (AICc) [44].

We investigated short–term increases or decreases in activity at camera stations with respect to the time attractant was placed by calculating the time elapsed since the attractant was replaced for each capture. Site visits were recorded as independent if at least 1 hour had elapsed since the previous capture of the same species at the same station [45]. To avoid peaks and valleys in visitation rates created by strongly diurnal or nocturnal species, we aggregated site visits into 24 h time periods representing bins of 1 day (0 h– 24 h) through 7 days (144 h– 168 h) after attractant was replaced. We restricted analysis to one week in order to focus on the short-term effects of the placement of attractant on each species. We calculated the observed probability of detecting each species for each of these 7 days by dividing the number of independent site visits for each 24 h time period by the total detections acquired. We then generated one thousand random capture histories of the same sample size as in the observed sample for each species using the following procedure: we first selected a survey station from the stations at which a given species was captured, we then selected a date from the dates the selected station was active, and a time from the species’ activity period probability density function, which was calculated using circular statistics in R package *activity* [46]. Finally, we calculated the time elapsed since attractant was refreshed for all random samples. We calculated the two-tailed probability of obtaining our observed sample based on the distribution of the random samples using a standard permutation test: (n + 1)/ N, where n is the number of random samples larger or smaller than the observed value, and N is the total number of expected values [47]. A significance level of α = 0.05 was used for all statistical tests. P-values < 0.025 indicated that sites were visited less often and p-values > 0.975 indicated that sites were visited more often than predicted in the random distribution, suggesting reduced and increased activity respectively.

## Results

### Rubbish pit survey

To assess scavenging behaviour, we surveyed rubbish pits for a cumulative total of 301 trap days, and obtained 187 independent captures of carnivores (Table 1). African civet and rusty-spotted genet were captured most often, and were photographed entering the pits. Marsh mongoose were detected 6 times, but were not observed entering the pits. African golden cat, serval, African palm civet, and servaline genet were never detected at rubbish pits. Giant forest rat, a potential African golden cat prey species, were also photographed entering rubbish pits on 27 occasions.

**Table 1:** Independent captures (> 1 h elapsed since the last capture of the same species), capture rates (per 100 trap days), naïve detection, and naïve occupancy of selected carnivore and prey species detected at 5 organic rubbish pits at the Makerere University Biological Field Station and during three systematic camera trap surveys conducted in 2013–2014 in Kibale National Park, Uganda.

Species	Rubbish pits		Systematic surveys			
	Photo events	Capture rate	Photo events	Capture rate	Naïve detection	Naïve occupancy
**Carnivores**						
African golden cat	0	0	201	2.06	0.037	0.543
Serval	0	0	36	0.37	0.019	0.189
African palm civet	0	0	136	1.40	0.030	0.433
African civet	38	12.62	433	4.45	0.077	0.496
Servaline genet	0	0	520	5.34	0.069	0.732
Rusty-spotted genet	62	20.6	713	7.32	0.118	0.480
Marsh mongoose	6	1.99	1796	18.45	0.181	0.874
**African golden cat prey**						
Blue duiker	0	0	484	4.97	0.074	0.622
Weyns’s red duiker	0	0	528	5.42	0.072	0.701
Giant forest rat	27	8.97	310	3.18	0.058	0.480

### Influence of attractant and effort on detection

Each survey lasted for 80–159 days, and cameras were left in place for 72.10 (SD 13.87) days, accruing a total of 9742 trap nights. We accumulated 3955 independent captures of the seven most commonly detected carnivore species, and 1348 captures of the three selected prey species (Table 1). Attractant was placed or refreshed 1,286 times across all stations.

**Fig 2:**
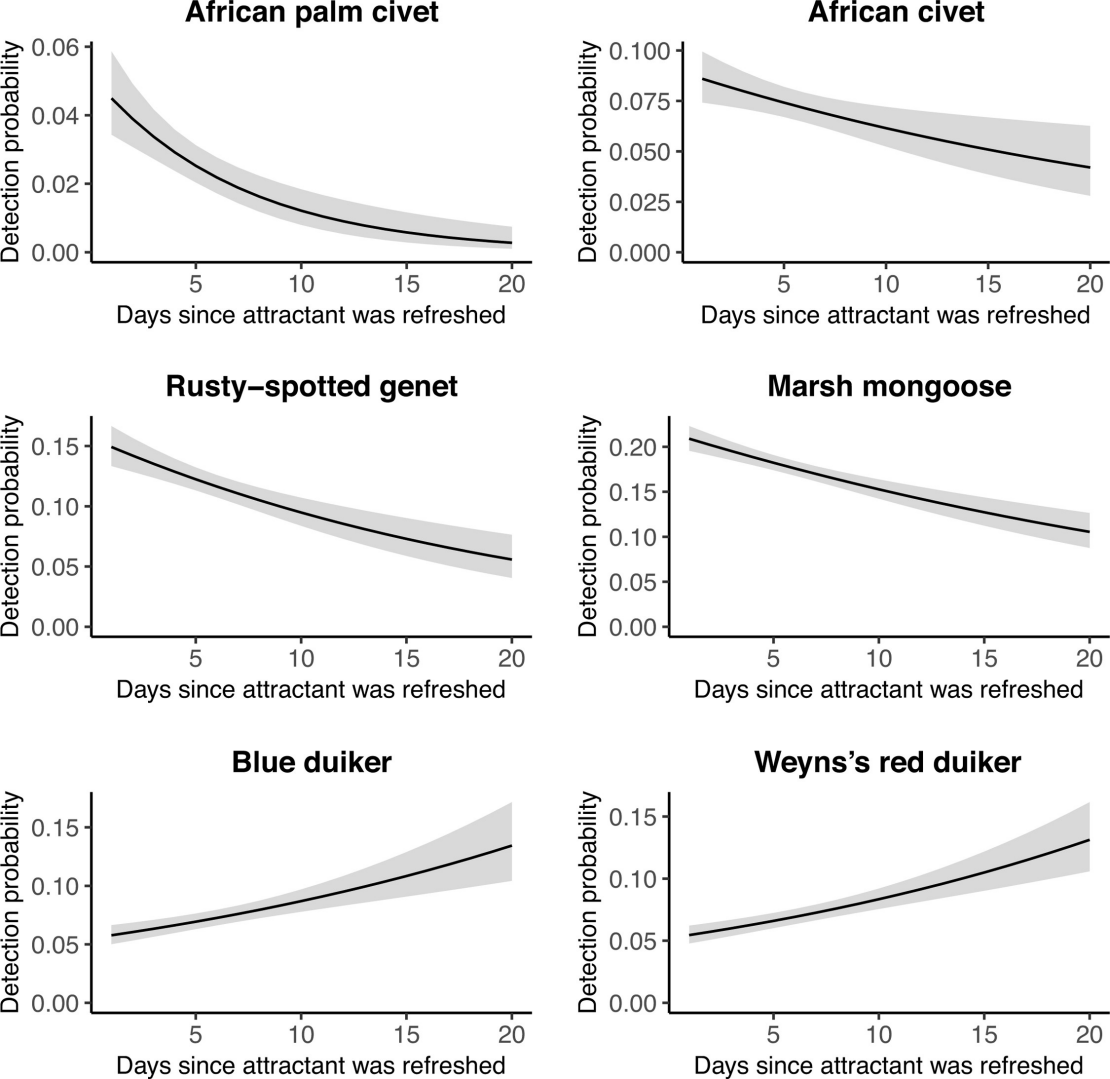
Predicted detection probabilities with respect to time since attractant was refreshed. Predictions are shown with 95% confidence intervals (grey bars) for four small carnivores and two duikers in Kibale National Park, Uganda, in 2013–2014. Only significant relationships are shown. Models for all species are available in S1 Fig.

**Fig 3:**
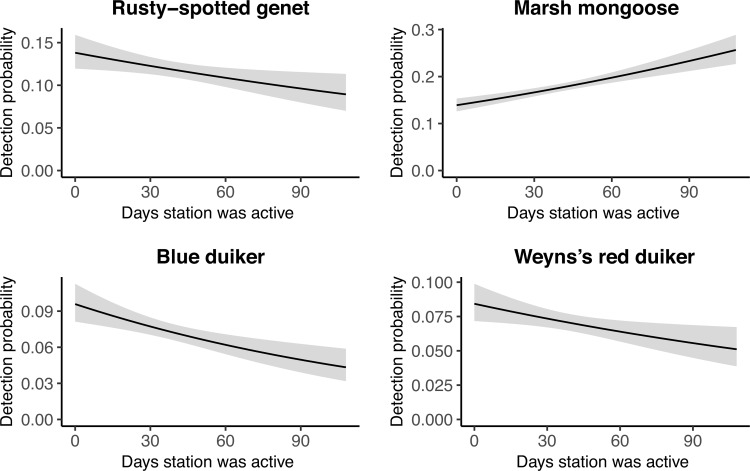
Predicted detection probabilities with respect to the time a camera remained active (incremental effort). Predictions are shown with 95% confidence intervals (grey bars) for two small carnivores and two duikers in Kibale National Park, Uganda, in 2013–2014. Only statistically significant relationships are shown. Models for all species are available in S3 Fig.

Immediately after attractants were replaced, detection probability was highest, followed by a significant decline, for African palm civet, African civet, rusty-spotted genet, and marsh mongoose and lowest, followed by a significant increase, for red duiker (Fig 2 and S1 Table and S1 Fig). African golden cat, servaline genet, blue duiker, and giant forest rat showed no attractant effect. At camera stations with higher effective trap days, detection probabilities of African golden cat, African civet, rusty-spotted genet, and marsh mongoose were lower (S1 Table and S2 Fig), which is possibly related to species distribution and habitat selection patterns. The incremental measure of effort, which calculated a new detection probability for each occasion based on the number of days an individual station had been active up to that occasion, significantly influenced detection of four species. Detection probability of rusty spotted genet, blue duiker, and red duiker decreased and detection probability of marsh mongoose increased the longer a station remained active (Figs 3 and S3).

### Short-term attractant effect

**Fig 4:**
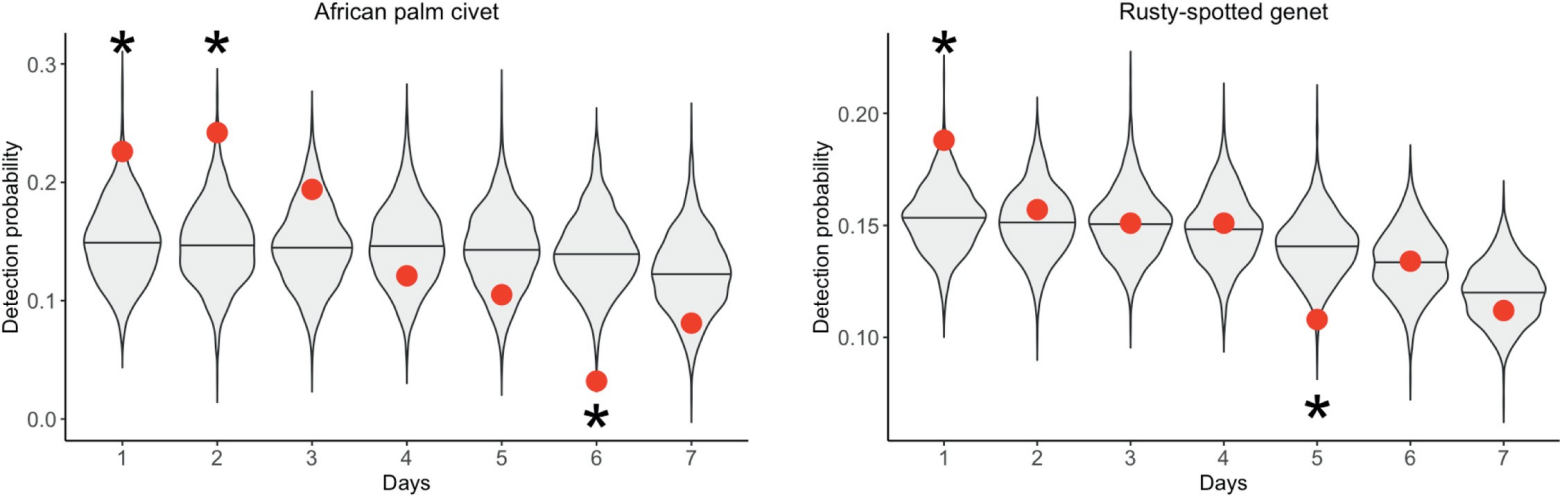
Increased detection probability following the replacement of attractant during three surveys in Kibale National Park, Uganda in 2013–2014. Violin plots represent the distribution of random detection probabilities. Observed detection probabilities are indicated with red dots. Significantly high and low detection probabilities are indicated with a star (α = 0.05).

**Fig 5:**
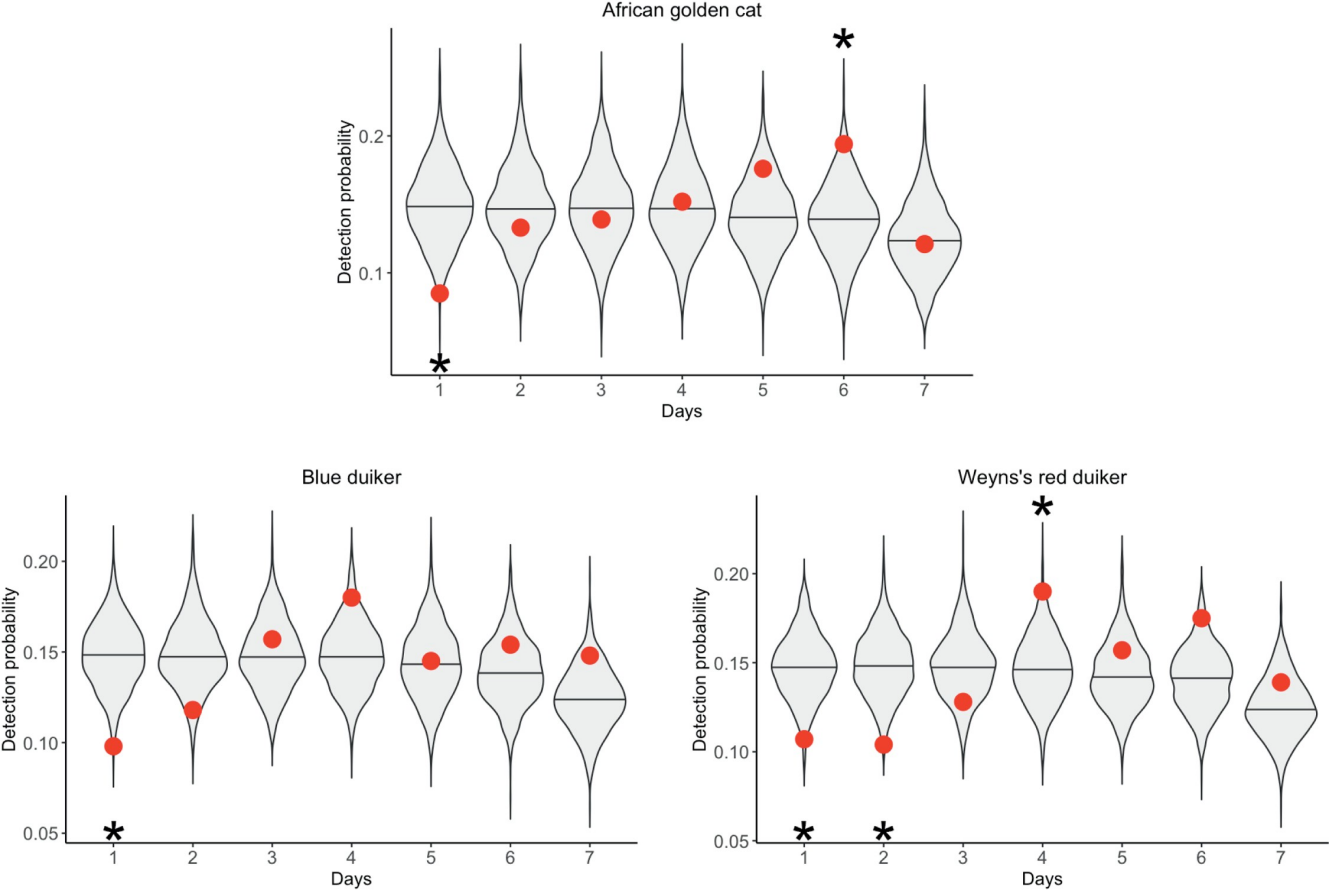
Decreased detection probability following the replacement of attractant during three surveys in Kibale National Park, Uganda in 2013–2014. Violin plots represent the distribution of random detection probabilities. Observed detection probabilities are indicated with red dots. Significantly high and low detection probabilities are indicated with a star (α = 0.05).

Rusty-spotted genet and African palm civet were more active than expected at stations within one day of refreshing attractant, indicating increased site use (Figs 4 and 5 and S2 Table and S4 Fig). African golden cat, blue duiker, and red duiker were less active than expected at stations within a day following refreshing of attractant, indicating decreased site use. These effects persisted for two days for African palm civet and red duiker.

## Discussion

We demonstrate that a complex of attractants that includes meat, fruit, and scent can be used to attract a community of small-bodied carnivores, including arboreal and semi-arboreal species. However, we show that detection probability of some species is negatively impacted by the placement of attractants. Additionally, we demonstrate that detection probability of some species changes over time as the attractant effect decays. When used sparingly, attractants can increase detection of animals that would normally avoid the trail or remain in the trees. This can expand the data typically gathered in large carnivore surveys to include a portion of the small carnivore guild, thus allowing guild level questions to be addressed. Additionally, it has been demonstrated elsewhere that increased sample sizes and additional individual recaptures improve the precision of analyses such as spatially explicit capture-recapture, thus contributing important data to small carnivore conservation [27, 28, 48, 49]. However, reduced detection probabilities of some species raise concerns for community studies. Those that maintain consistent attractant freshness by renewing baits and lures every few days may significantly reduce site use of a variety of species, even target species like African golden cats in this study.

Our results corroborate other studies that showed prey species avoid stations baited for carnivores [33]. Both blue and red duiker were less likely to be photographed in the 24 h following placement of attractant, and their detection probabilities increased as time elapsed since attractant was refreshed. Increased activity of carnivores due to the presence of attractants may unintentionally manipulate the “landscape of fear” [50]. Alterations to existing risk dynamics may temporarily reduce detection probability of the prey species [33, 51, 52]. However, predators also follow prey movements [53]. In this study, both golden cat and blue duiker, which are an important golden cat prey [38], were less active at camera stations immediately after attractant was replaced.

Several studies have tested the effect of attractants using a control period without attractants and a treatment period with attractants in a single survey [21, 26]. They have provided important information on the overall impact of attractants on individual species and communities [27, 28]. Since we were focusing on the short-term impact of attractant placement on detection probabilities of a variety of species, we used the same treatment at all stations across all three surveys. In doing so, we collected a relatively large dataset where attractant was placed 1,286 times, which provided a much higher power to detect changes in detection than if we had conducted a special, shorter survey to specifically test attractants. We also did not parse the effect of meat, fruit, and scent, as this has also been well studied [26, 27]. Testing different combinations of attractants introduces variation that must be accounted for in common analyses such as occupancy modelling [41]. We acknowledge that in some cases, it may be important to know which attractants had stronger effects on each species. Indeed, this could be an important avenue of further inquiry.

An important confounding factor that must be carefully considered is that attractants were combined with our human scent. Some species are tolerant of, and even seek out, areas used by humans [54], while other species are more sensitive to human presence, particularly those targeted by bushmeat hunting, such as duikers [55, 56]. In fact, it could be that species not positively influenced by attractants are repelled by the presence of humans. This effect could also be cumulative, which could explain why detection rates gradually declined for both duiker species the longer a camera remained active. There may be a species-specific threshold in the continuum between food reward or attraction to lure and the fear human scent that must be overcome in order to avoid deterring target species, as occurred here with African golden cats. It should also be noted that we do not have evidence that any species increased or decreased use of the greater area around camera stations, only that they altered their use of the particular section of trail covered by the camera. It is possible that these species with lower detection probabilities were still active in the area, but were not using the trail [23].

Our final caveat is that placement of attractants on the ground may be causing them to unnaturally expose themselves to interference competition by using trails typically monopolised by larger competitors [23]. While the amount of consumable bait was small, it is possible that some individuals included areas with camera stations into their foraging more often than they normally would. Therefore, while raw capture rates are never a statistically robust measure, it is particularly important to avoid using them to make comparisons when attractants are used [57].

Attractants are an effective tool for increasing detection of elusive carnivores [28]. However, we have highlighted some important shortcomings that may discourage their use in some cases. Our results suggest that species do exhibit short-term changes in detectability following the placement of attractants. Most importantly, response to attractants was species-specific and not necessarily straightforward and predictable. As ecological studies shift from single species to guild and community studies [58–60], it is important to consider that the use of attractants can elicit fine-scale changes in camera station use that may influence the robustness of some community level conclusions. Analyses involving multispecies comparisons of spatiotemporal partitioning [14] or predator-prey interactions [61] should account for such effects. Specifically, we recommend that, where attractants are used in surveys intended for occupancy modelling, occasion length should equal the length of time between the refreshing of attractants in order to mask this variation in detection probability that occurs over time. Additionally, studies often refresh bait frequently [28, 30]. Given the short-term reductions in activity levels demonstrated here, studies that refresh bait every 2–4 d may not allow capture rates to return to background levels. This would create a stable, but continuously inflated detection probability for some species, and suppressed detection probability for others. It is therefore important to clearly define the desired outcomes of a survey, and to carefully weigh the pros and cons of each sampling design, including the use or omission of attractants, as it has been suggested in other studies [17, 21, 29]. In order to minimise bias for occupancy and density analysis, attractant protocols should ensure that the attractant effect area (scent transmission distance) is as small as possible and certainly significantly smaller than target species’ activity areas (home ranges) to avoid altering the biology of individual species and community dynamics.

## Conclusions

Small forest carnivores are notoriously elusive and difficult to study. While the benefits of using attractant, particularly edible bait, in large carnivore surveys has been questioned [29], this study shows that attractants could be an important tool for achieving robust sample sizes of small-bodied mammals. As camera trap technology has advanced and become more affordable, small carnivores have become the focus of intensive scientific inquiry [62–64]. Our study confirms that the use of attractants can significantly improve capture rates of elusive small carnivores, which are often under-studied and data deficient [65]. However, we also demonstrated that some species respond adversely to the presence of attractant, or possibly the presence of humans placing attractant, and temporarily avoid camera stations. It is therefore important to strike a balance between increasing detection for some species and avoiding human presence that might deter others. Even though reduced activity is immediate in some species, we also demonstrate that this effect lasts only 1–2 days before levelling off. Given these results, accurate reporting of camera trap protocols is particularly important in the era of big data, where camera trap data from a variety of locations are being used to ask questions about species that were not originally the targets of the survey [66]. While the use of attractant can increase our understanding of species that have not traditionally been the primary focus of research, practitioners should consider that some species may have lower detection probabilities or reduced activity when attractants are deployed before making inferences about their ecological and conservation requirements.

## Supporting information

S1 Table Full model sets exploring the influence of attractant freshness, the total length a station was left in place, and an incremental measure of how long a station had been active on each occasion. In order to understand how attractants influenced detection in the short-term, we used an occasion length of 1 day. We tested the influence of these covariates on seven small carnivores and three prey species in Kibale National Park, Uganda, in 2013–2014.
(PDF)

S2 Table Short term changes in detection following placement of attractants for seven carnivores and three prey species documented during three surveys in Kibale National Park, Uganda in 2013–2014. Detection rates that differ significantly from random predictions are highlighted in bold. P-values < 0.05 suggest reduced station use and values > 0.95 suggest increased station use. We restricted analysis to 7 days after replacement of attractants in order to focus on short term responses.
(PDF)

S1 Fig A: Plots showing the relationship between detection probability and attractant freshness for seven small carnivores in Kibale National Park, Uganda, in 2013–2014. B: Plots showing the relationship between detection probability and attractant freshness for three African golden cat prey species in Kibale National Park, Uganda, in 2013–2014.
(PDF)

S2 Fig A: Plots showing the relationship between detection probability and total effort for seven small carnivores in Kibale National Park, Uganda, in 2013–2014. B: Plots showing the relationship between detection probability and total effort for three Afrcican golden cat prey species in Kibale National Park, Uganda, in 2013–2014.
(PDF)

S3 Fig A: Plots showing the relationship between detection probability and incremental effort (the number of days a station has been active on each consecutive day) for seven small carnivores in Kibale National Park, Uganda, in 2013–2014. B: Plots showing the relationship between detection probability and incremental effort (the number of days a station has been active on each consecutive day) for three African golden cat prey species in Kibale National Park, Uganda, in 2013–2014.
(PDF)

S4 Fig Detection probability of (A) seven small carnivores and (B) three African golden cat prey species following the replacement of attractant during three surveys in Kibale National Park, Uganda in 2013–2014. A significance level of 0.05 was used for all statistical tests. Violin plots represent the distribution of random detection probabilities. Observed detection probabilities are indicated with red dots. Detection probabilities that deviate significantly from the random distribution are indicated with a star.
(PDF)

S1 Dataset The full dataset of captures and bait placement used for all analyses in this manuscript. (ZIP)
