# Supplementary material for: Maximising camera trap data: Using attractants to improve detection of elusive species in multi-species surveys
Source: PLoS One. 2019 May 29;14(5):e0216447. doi: 10.1371/journal.pone.0216447 (PMC6541258; doi:10.1371/journal.pone.0216447)
Supplement: S1 Table — In order to understand how attractants influenced detection in the short-term, we used an occasion length of 1 day. We tested the influence of these covariates on seven small carnivores and three prey species in Kibale National Park, Uganda, in 2013–2014. (PDF) [file pone.0216447.s001.pdf]

S1 Table. Full model sets exploring the influence of attractant freshness, the total length a station was left in place, and an incremental measure of how long a station had been active on each occasion. In order to understand how attractants influenced detection in the short-term, we used an occasion length of 1 day. We tested the influence of these covariates on seven small carnivores and three prey species in Kibale National Park, Uganda, in 2013 – 2014.

Detection covariates	K	AICc	ΔAICc	ModelLik	AICcWt	LL
**African golden cat**						
Total effort	3	1812.94	0.000	1.000	0.457	-903.37
Null	2	1814.70	1.760	0.415	0.189	-905.30
Attractant + Total effort	4	1815.06	2.128	0.345	0.158	-903.37
Incremental effort	3	1816.05	3.116	0.211	0.096	-904.93
Attractant	3	1816.77	3.837	0.147	0.067	-905.29
Attractant + Incremental effort	4	1818.18	5.243	0.073	0.033	-904.93
**Serval**						
Null	2	446.38	0.000	1.000	0.411	-221.14
Incremental effort	3	447.99	1.611	0.447	0.184	-220.90
Attractant	3	448.45	2.065	0.356	0.147	-221.13
Total effort	3	448.48	2.097	0.350	0.144	-221.14
Attractant + Incremental effort	4	450.12	3.735	0.155	0.064	-220.89
Attractant + Total effort	4	450.58	4.197	0.123	0.050	-221.13
**African palm civet**						
Attractant	3	1293.22	0.000	1.000	0.464	-643.51
Attractant + Total effort	4	1293.67	0.449	0.799	0.370	-642.67
Attractant + Incremental effort	4	1295.28	2.058	0.357	0.166	-643.47
Total effort	3	1318.64	25.420	0.000	0.000	-656.22
Null	2	1320.46	27.246	0.000	0.000	-658.18
Incremental effort	3	1322.48	29.258	0.000	0.000	-658.14
**African civet**						
Attractant + Total effort	4	2760.15	0.000	1.000	0.753	-1375.91
Attractant	3	2764.04	3.896	0.143	0.107	-1378.92
Total effort	3	2764.54	4.390	0.111	0.084	-1379.17
Attractant + Incremental effort	4	2765.52	5.376	0.068	0.051	-1378.60
Null	2	2770.83	10.682	0.005	0.004	-1383.37
Incremental effort	3	2772.73	12.582	0.002	0.001	-1383.27

Detection covariates	K	AICc	ΔAICc	ModelLik	AICcWt	LL
**Servaline genet**						
Null	2	3732.56	0.000	1.000	0.305	-1864.23
Total effort	3	3733.24	0.674	0.714	0.218	-1863.52
Incremental effort	3	3733.63	1.062	0.588	0.180	-1863.72
Attractant	3	3734.25	1.686	0.430	0.131	-1864.03
Attractant + Increm effort	4	3735.16	2.596	0.273	0.083	-1863.42
Attractant + Total effort	4	3735.19	2.621	0.270	0.082	-1863.43
**Rusty-spotted genet**						
Attractant + Total effort	4	3601.81	0.000	1.000	1.000	-1796.74
Total effort	3	3619.71	17.896	0.000	0.000	-1806.76
Attractant + Incremental effort	4	3628.05	26.239	0.000	0.000	-1809.86
Attractant	3	3629.27	27.464	0.000	0.000	-1811.54
Incremental effort	3	3652.32	50.512	0.000	0.000	-1823.06
Null	2	3656.32	54.510	0.000	0.000	-1826.11
**Marsh mongoose**						
Attractant + Total effort	4	7943.73	0.000	1.000	0.997	-3967.70
Attractant + Incremental effort	4	7955.39	11.654	0.003	0.003	-3973.53
Total effort	3	7969.27	25.532	0.000	0.000	-3981.54
Attractant	3	7996.99	53.258	0.000	0.000	-3995.40
Incremental effort	3	8002.40	58.670	0.000	0.000	-3998.10
Null	2	8035.65	91.915	0.000	0.000	-4015.78
**Blue duiker**						
Attractant + Incremental effort	4	3303.02	0.000	1.000	0.999	-1647.34
Attractant	3	3318.53	15.510	0.000	0.000	-1656.17
Attractant + Total effort	4	3320.06	17.046	0.000	0.000	-1655.87
Incremental effort	3	3325.91	22.899	0.000	0.000	-1659.86
Null	2	3337.18	34.163	0.000	0.000	-1666.54
Total effort	3	3337.65	34.637	0.000	0.000	-1665.73
**Red duiker**						
Attractant + Incremental effort	4	3649.81	0.000	1.000	0.935	-1820.74
Attractant + Total effort	4	3655.56	5.751	0.056	0.053	-1823.61
Attractant	3	3658.48	8.672	0.013	0.012	-1826.14
Incremental effort	3	3682.37	32.564	0.000	0.000	-1838.09
Null	2	3686.34	36.534	0.000	0.000	-1841.12
Total effort	3	3687.43	37.629	0.000	0.000	-1840.62

Detection covariates	K	AICc	ΔAICc	ModelLik	AICcWt	LL
**Giant forest rat**						
Null	2	2368.68	0.000	1.000	0.259	-1182.29
Attractant	3	2368.87	0.192	0.909	0.235	-1181.34
Incremental effort	3	2369.73	1.053	0.591	0.153	-1181.77
Attractant + Total effort	4	2370.11	1.433	0.489	0.126	-1180.89
Attractant + Incremental effort	4	2370.22	1.539	0.463	0.120	-1180.95
Total effort	3	2370.45	1.766	0.414	0.107	-1182.13
