# Supplementary material for: Maximising camera trap data: Using attractants to improve detection of elusive species in multi-species surveys
Source: PLoS One. 2019 May 29;14(5):e0216447. doi: 10.1371/journal.pone.0216447 (PMC6541258; doi:10.1371/journal.pone.0216447)
Supplement: S2 Table — Detection rates that differ significantly from random predictions are highlighted in bold. P-values < 0.05 suggest reduced station use and values > 0.95 suggest increased station use. We restricted analysis to 7 days after replacement of attractants in order to focus on short term responses. (PDF) [file pone.0216447.s002.pdf]

S2 Table. Short term changes in detection following placement of attractants for seven carnivores and three prey species documented during three surveys in Kibale National Park, Uganda in 2013 - 2014. Two -tailed probability was calculated using a standard permutation test. Detection rates that differ significantly from random predictions are highlighted in bold. P-values < 0.025 suggest reduced station use and values > 0.975 suggest increased station use. We restricted analysis to 7 days after replacement of attractants in order to focus on short term responses.

	Day 1	Day 2	Day 3	Day 4	Day 5	Day 6	Day 7
**African golden cat**							
Photographic events	14	22	23	25	29	32	20
Observed det prob	0.085	0.133	0.139	0.152	0.176	0.194	0.121
Random det prob	0.149	0.148	0.148	0.148	0.142	0.139	0.125
p-value	**0.004**	0.298	0.379	0.564	0.889	**0.971**	0.451
**Serval**							
Photographic events	5	6	6	1	4	3	2
Observed det prob	0.185	0.222	0.222	0.037	0.148	0.111	0.074
Random det prob	0.151	0.152	0.149	0.146	0.144	0.133	0.124
p-value	0.704	0.847	0.835	0.030	0.534	0.372	0.197
**African palm civet**							
Photographic events	28	30	24	15	13	4	10
Observed det prob	0.226	0.242	0.194	0.121	0.105	0.032	0.081
Random det prob	0.150	0.148	0.146	0.147	0.143	0.141	0.123
p-value	**0.987**	**0.995**	0.911	0.226	0.126	**0.001**	0.092
**African civet**							
Photographic events	52	60	62	50	54	33	34
Observed det prob	0.151	0.174	0.18	0.145	0.157	0.096	0.099
Random det prob	0.151	0.150	0.150	0.148	0.142	0.138	0.121
p-value	0.495	0.886	0.931	0.440	0.782	**0.013**	0.093
**Servaline genet**							
Photographic events	45	70	55	67	48	50	48
Observed det prob	0.117	0.183	0.144	0.175	0.125	0.131	0.125
Random det prob	0.148	0.148	0.149	0.146	0.144	0.138	0.126
p-value	0.041	0.961	0.391	0.942	0.138	0.312	0.453

	Day 1	Day 2	Day 3	Day 4	Day 5	Day 6	Day 7
**Rusty-spotted genet**							
Photographic events	111	93	89	89	64	79	66
Observed det prob	0.188	0.157	0.151	0.151	0.108	0.134	0.112
Random det prob	0.154	0.152	0.151	0.149	0.141	0.134	0.120
p-value	**0.989**	0.628	0.496	0.558	**0.01**	0.497	0.274
**Marsh mongoose**							
Photographic events	213	237	250	228	207	183	161
Observed det prob	0.144	0.16	0.169	0.154	0.14	0.124	0.109
Random det prob	0.149	0.148	0.147	0.147	0.144	0.141	0.124
p-value	0.291	0.886	**0.986**	0.737	0.314	0.027	0.055
**Blue duiker**							
Photographic events	33	40	53	61	49	52	50
Observed det prob	0.098	0.118	0.157	0.180	0.145	0.154	0.148
Random det prob	0.148	0.149	0.148	0.148	0.144	0.139	0.124
p-value	**0.003**	0.044	0.683	0.955	0.527	0.802	0.911
**Red duiker**							
Photographic events	36	35	43	64	53	59	47
Observed det prob	0.107	0.104	0.128	0.190	0.157	0.175	0.139
Random det prob	0.147	0.148	0.148	0.147	0.143	0.141	0.125
p-value	**0.014**	**0.006**	0.118	**0.991**	0.781	0.969	0.803
**Giant forest rat**							
Photographic events	43	44	41	35	29	22	26
Observed det prob	0.179	0.183	0.171	0.146	0.121	0.092	0.108
Random det prob	0.148	0.147	0.147	0.145	0.144	0.141	0.128
p-value	0.899	0.931	0.853	0.500	0.159	**0.012**	0.187
