# Supplementary material for: Maximising camera trap data: Using attractants to improve detection of elusive species in multi-species surveys
Source: PLoS One. 2019 May 29;14(5):e0216447. doi: 10.1371/journal.pone.0216447 (PMC6541258; doi:10.1371/journal.pone.0216447)

S3 Fig A. Plots showing the relationship between detection probability and incremental effort (the number of days a station has been active on each consecutive day) for seven small carnivores in Kibale National Park, Uganda, in 2013 – 2014.

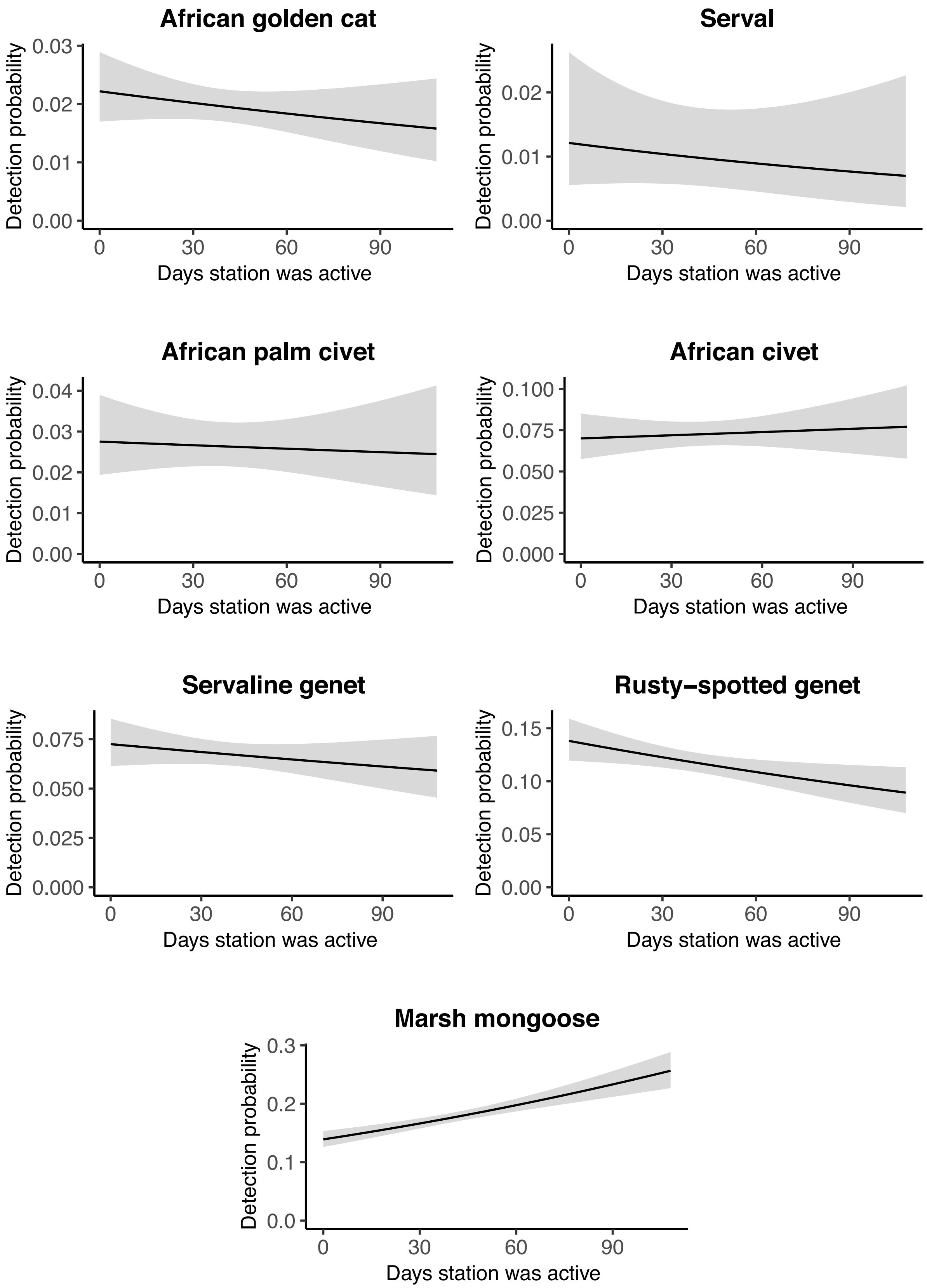

S4 Fig B. Plots showing the relationship between detection probability and incremental effort (the number of days a station has been active on each consecutive day) for three African golden cat prey species in Kibale National Park, Uganda, in 2013 – 2014.

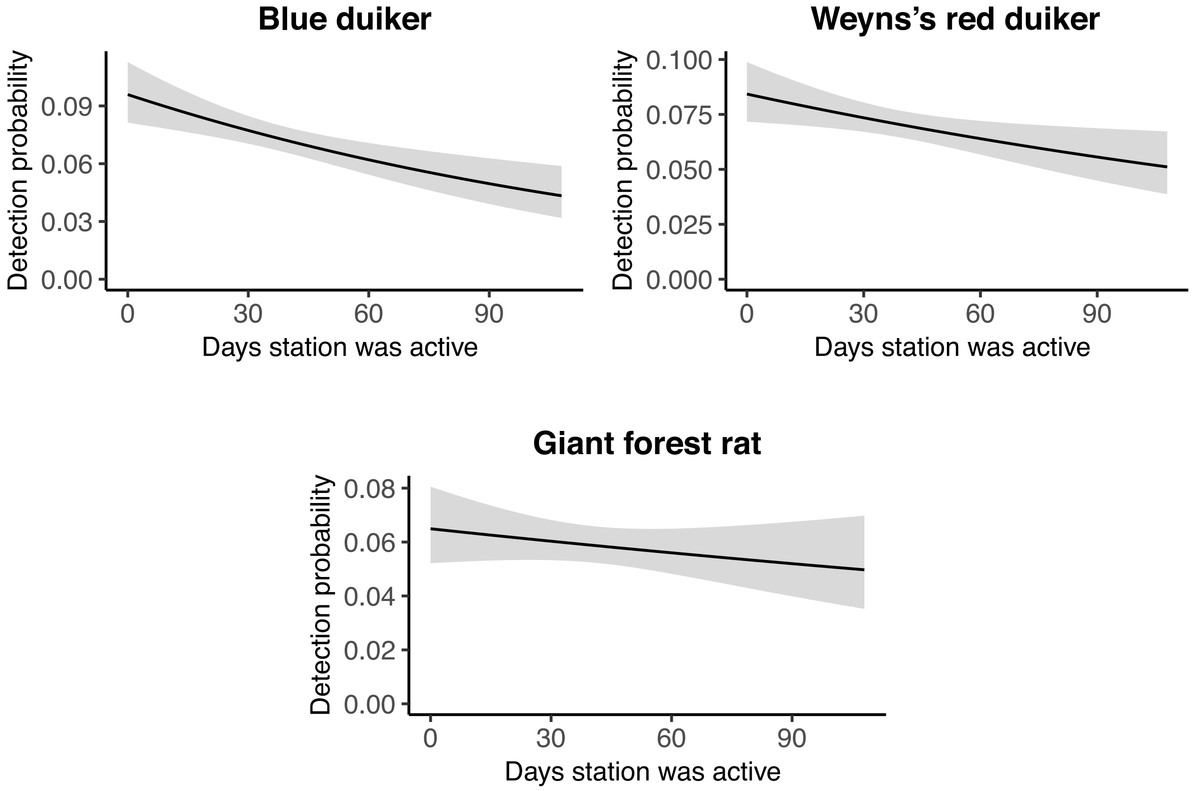

Supplement: S3 Fig — A: Plots showing the relationship between detection probability and incremental effort (the number of days a station has been active on each consecutive day) for seven small carnivores in Kibale National Park, Uganda, in 2013–2014. B: Plots showing the relationship between detection probability and incremental effort (the number of days a station has been active on each consecutive day) for three African golden cat prey species in Kibale National Park, Uganda, in 2013–2014. (PDF) [file pone.0216447.s005.pdf]
