# Supplementary material for: Maximising camera trap data: Using attractants to improve detection of elusive species in multi-species surveys
Source: PLoS One. 2019 May 29;14(5):e0216447. doi: 10.1371/journal.pone.0216447 (PMC6541258; doi:10.1371/journal.pone.0216447)

S4 Fig A. Detection probability of seven carnivores following the replacement of attractant during three surveys in Kibale National Park, Uganda in 2013 – 2014. Violin plots represent the distribution of random detection probabilities. Observed detection probabilities are indicated with red dots. Detection probabilities that deviate significantly from the random distribution are indicated with a star. A significance level of 0.05 was used for all statistical tests.

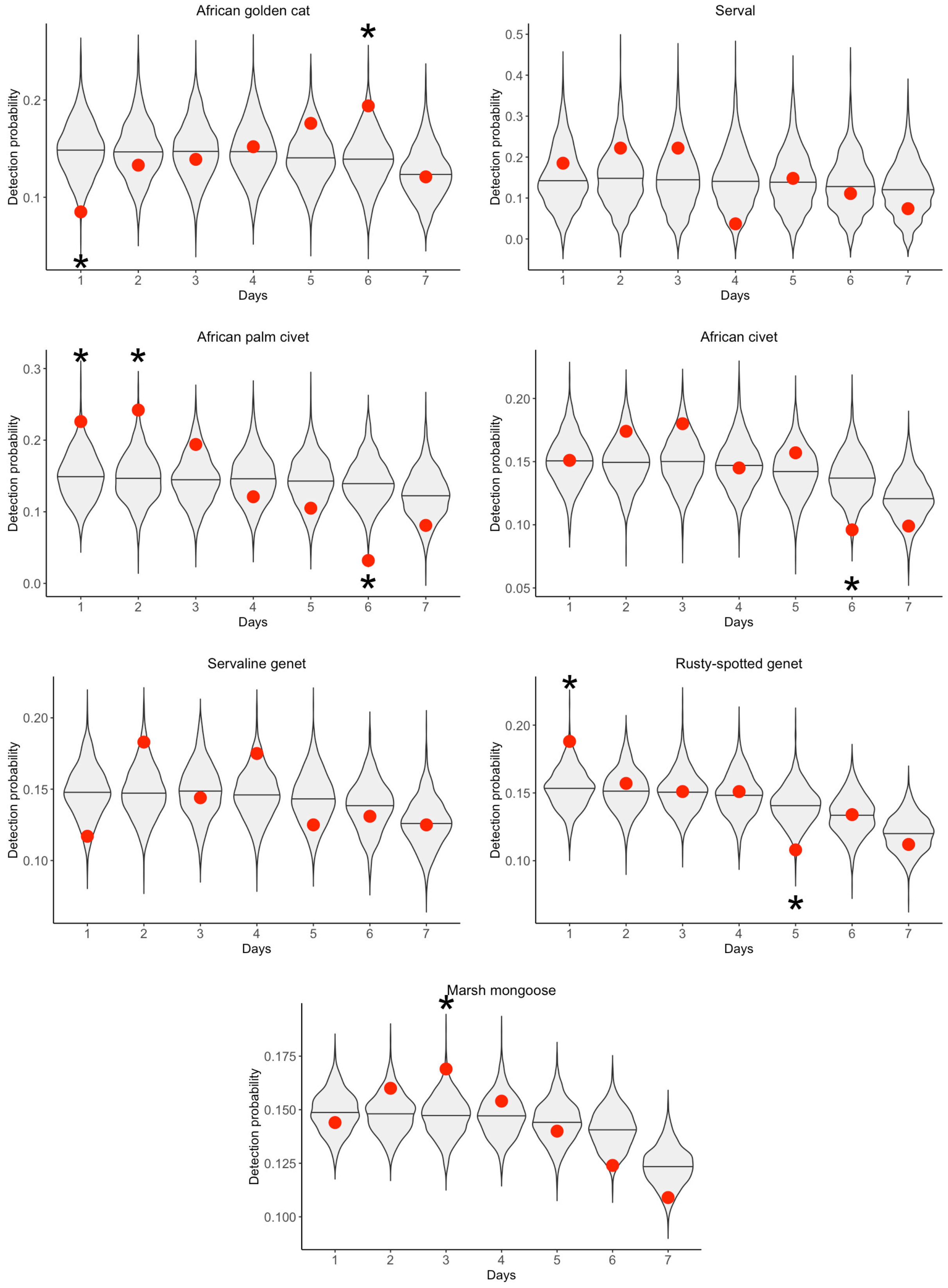

S6 Fig B. Detection probability of three African golden cat prey species following the replacement of attractant during three surveys in Kibale National Park, Uganda in 2013 – 2014. A significance level of 0.05 was used for all statistical tests. Violin plots represent the distribution of random detection probabilities. Observed detection probabilities are indicated with red dots. Detection probabilities that deviate significantly from the random distribution are indicated with a star.

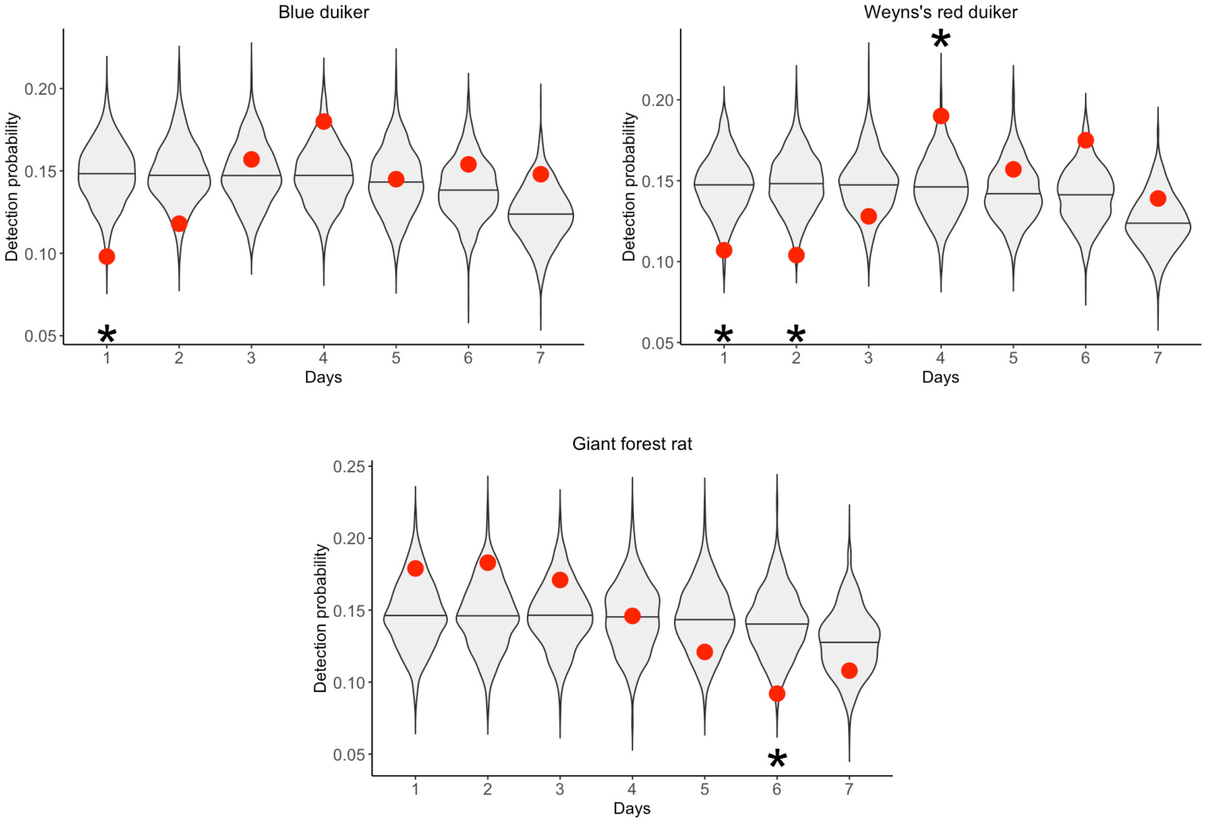

Supplement: S4 Fig — Detection probability of (A) seven small carnivores and (B) three African golden cat prey species following the replacement of attractant during three surveys in Kibale National Park, Uganda in 2013–2014. A significance level of 0.05 was used for all statistical tests. Violin plots represent the distribution of random detection probabilities. Observed detection probabilities are indicated with red dots. Detection probabilities that deviate significantly from the random distribution are indicated with a star. (PDF) [file pone.0216447.s006.pdf]
